# Small Peptide Ligands for Targeting EGFR in Triple Negative Breast Cancer Cells

**DOI:** 10.1038/s41598-019-38574-y

**Published:** 2019-02-25

**Authors:** Hanieh Hossein-Nejad-Ariani, Emad Althagafi, Kamaljit Kaur

**Affiliations:** Chapman University School of Pharmacy (CUSP), Harry and Diane Rinker Health Science Campus, Chapman University, Irvine, California 92618-1908 USA

## Abstract

The efficacy of chemotherapy for cancer treatment can be increased by targeted drug delivery to the cancer cells. This is particularly important for triple negative breast cancer (TNBC) for which chemotherapy is a major form of treatment. Here we designed and screened a library of 30 peptides starting with a previously reported epidermal growth factor receptor (EGFR) targeting peptide GE11 (YHWYGYTPQNVI). A direct peptide array-whole cell binding assay, where the peptides are conjugated to a cellulose membrane, was used to identify four peptides with enhanced binding to TNBC cells. Next, the four peptides were synthesized as FITC-labelled soluble peptides to study their direct uptake by TNBC cells using flow cytometry. The results showed that peptide analogue **22** had several fold higher uptake by the TNBC cells compared to the lead peptide GE11. The specific uptake of the peptide analogue **22** was confirmed by competition experiment using pure EGF protein. Further, peptide **22** showed dose dependent uptake by the TNBC MDA-MB-231 cells (10^5^) with uptake saturating at around 2 μM peptide concentration. Thus, peptide **22** is a promising EGFR specific TNBC cell binding peptide that can be conjugated directly to a chemotherapeutic drug or to nanoparticles for targeted drug delivery to enhance the efficacy of chemotherapy for TNBC treatment.

## Introduction

Triple negative breast cancer (TNBC) is an important subtype of breast cancer as it is more aggressive than other subtypes and has poor prognosis^1–4^. TNBC is characterized as a breast cancer phenotype that is estrogen receptor (ER) negative, progesterone receptor (PR) negative, and is without the overexpression of human epidermal growth factor receptor 2 (HER2), and hence is called the “triple-negative” phenotype^2^. TNBC is more common in younger women, premenopausal African American women, and women of lower socioeconomic status (SES) when compared to other subtypes. Diagnosis of TNBC usually happens at the more advanced stage of the disease and it usually leads to death within the first five years of diagnosis. Additionally, in TNBC, high grade rapidly growing tumors are poorly differentiated and metastasis tends to occur in soft tissues including lungs and brain rather than in bone^4^. Chemotherapy is a major way to treat TNBC. However it has been difficult to target chemotherapy to TNBC due to the lack of well-defined molecular targets^2,5,6^.

Peptide **1** (or GE11, Table 1) is a dodecapeptide that binds specifically to epidermal growth factor receptor (EGFR or ErbB1) overexpressed in a number of tumors of epithelial origin including breast cancer, and is being used as a cancer cell targeting peptide^7–10^. EGFR has been suggested to play an important role in TNBC^11,12^. Several TNBC cell lines, like MDA-MB-468 and MDA-MB-231, show high expression levels of EGFR^13,14^. Although therapeutic monoclonal antibodies like cetuximab and tyrosine kinase inhibitors like lapatinib have not shown much success in clinical trials for TNBC patients, several EGFR-targeted antibody-drug conjugates (ADCs) show great potential for TNBC treatment and are currently undergoing clinical trials for TNBC patients^11^. Similarly peptide-drug conjugates can be designed for targeting EGFR in TNBC. The cancer cell targeting peptides are not therapeutic like antibodies, but have several advantages as targeting ligands over antibodies such as they can be easily synthesized, are more economical, can be designed as stable analogues, and display low immunogenicity.

**Table 1:** Designed peptide GE11 (or peptide **1**) library and the relative cell adhesion ratio of the peptides.

Peptide#	Sequence	Relative Cell Adhesion			
		MDA-MB-468	MDA-MB-231	MDA-MB-436	MCF-10A
1	YHWYGYTPQNVI	1.0	1.0	1.0	1.0
2	WQTNYIHPYVYG	1.1	1.2	1.1	1.0
3	YGPWYNHYITQV	1.0	1.1	1.1	1.0
4	VPWXEPAYQRFL	1.1	1.3	1.1	1.2
5	WXEAAYQRFL	1.3	1.7	1.8	1.7
6	**A**HWYGYTPQNVI	1.2	1.3	1.0	1.1
7	Y**A**WYGYTPQNVI	1.1	1.1	1.0	1.0
8	YH**A**YGYTPQNVI	0.8	1.0	0.9	0.9
9	YHW**A**GYTPQNVI	1.3	1.4	1.2	1.0
10	YHWY**A**YTPQNVI	1.3	1.5	1.2	1.1
11	YHWYG**A**TPQNVI	1.2	1.3	1.0	1.0
12	YHWYGY**A**PQNVI	1.0	1.3	1.0	1.1
13	YHWYGYT**A**QNVI	1.3	1.5	1.1	1.1
14	YHWYGYTP**A**NVI	1.4	1.7	1.2	1.2
15	YHWYGYTPQ**A**VI	1.2	1.6	0.9	1.1
16	YHWYGYTPQN**A**I	1.1	1.4	1.0	1.2
17	YHWYGYTPQNV**A**	1.4	1.6	1.2	1.2
18	YHWYGYTPQNV	1.4	1.9	1.3	1.2
19	YHWYGYTPQN	1.3	2.0	1.1	1.1
20	HWYGYTPQNVI	1.2	1.5	1.0	1.2
21	WYGYTPQNVI	1.4	1.9	1.0	1.2
22	YHWYGYTP**E**NVI	1.6	2.6	1.3	1.2
23	YHWYGYTPQ**D**VI	1.6	2.8	1.0	1.4
24	Y**K**WYGYTPQNVI	1.1	1.4	1.0	1.0
25	Y**R**WYGYTPQNVI	1.4	1.9	1.0	1.2
26	YHWYGYTP**K**NVI	1.3	1.6	1.0	0.8
27	YHWYGYTPQ**K**VI	1.2	1.8	1.0	1.0
28	**F**HWYGYTPQNVI	1.2	1.5	0.8	1.2
29	YHW**F**GYTPQNVI	1.2	1.7	1.1	1.1
30	YHWYG**F**TPQNVI	1.3	1.5	0.9	1.0

The letter X in the peptide sequence represents the amino acid norleucine (Nle).

Several peptides that target breast cancer cells have been engineered for enhanced specificity and proteolytic stability for clinical applications^15–17^. Peptides like RGD^18^, NGR^19–21^, and p160 (VPWMEPAYQRFL)^22–24^ target a number of different receptors such as integrin, aminopeptidase N (or CD13) and keratin 1, respectively, overexpressed by breast cancer cells. A cyclic analogue of peptide p160, N- to C terminal cyclized WXEAAYQkFL (where k is D-lysine), was recently reported that preferentially accumulates in tumor after injection in mice carrying orthotopic breast MDA-MB-231 tumors^25^. Similarly, peptide **1** is a good candidate peptide for breast cancer targeting as it binds EGFR, and can be engineered for proteolytically stability and specific targeting of TNBC cells. Peptide **1** was identified by screening a phage display peptide library against purified human EGFR protein^8^. The peptide is internalized by cells overexpressing EGFR and when delivered i.v. in mice it accumulates in EGFR overexpressing tumor xenografts. Peptide **1** has been used as the targeting ligand for delivery of genes using polyethylenimine (PEI) polyplexes and antisense oligonucleotide nanoparticles^8,26^. A number of studies have explored peptide **1** as a targeting ligand in nanoparticles to treat cancers including breast and ovarian malignant cells^27–31^.

Here we have designed a synthetic peptide library of 29 analogues of peptide **1** (GE11)^8^ with an aim to target EGFR specifically in TNBC cells (Table 1). The library was synthesized directly on cellulose membrane where the peptides remain covalently conjugated to the membrane. The library was screened against TNBC cells by incubating the membrane with different cells followed by labeling the bound cells with a fluorescent dye. Peptides showing highest relative fluorescence compared to the lead peptide (peptide **1**) were selected from the whole cell-peptide binding assay, and were individually synthesized as well as their uptake in normal and TNBC cells was compared. Peptide **4** (or p160) reported previously to target breast cancer cells was used as a positive control peptide^23–25^. Several new analogues of peptide **1** with higher binding (using whole cell-peptide binding assay) and uptake (using flow cytometry) to TNBC were identified. The new analogues of peptide **1** that target EGFR and specifically bind TNBC cells can be used for targeted delivery of chemotherapeutics like doxorubicin for TNBC treatment.

## Results and Discussion

### Peptide Array Synthesis

A peptide array consisting of 30 peptides in duplicates (60 spots) was synthesized on functionalized cellulose membrane (amino-PEG_500_) using automated spot synthesis ResPep SL apparatus. The free amino functional group present on the surface of the membrane was used to covalently couple the first amino acid, and thereafter the remaining amino acids were coupled sequentially to complete the sequences. Peptide array was synthesized using 96 spot mode (12 columns and 8 rows). Each peptide was synthesized in duplicates and a spot was skipped in order to separate from the next peptide sequence.

### Peptide Library

Peptide **1** (GE11, Table 1) is a 12-mer peptide rich in hydrophobic aromatic amino acids (three tyrosine’s and one tryptophan). Using previous strategy^21,32^, we designed a library consisting of GE11-derived peptide sequences. The library of 30 peptides includes negative (**2** and **3**, scrambled) and positive (**4** or peptide p160 and **5** or peptide 18 from a previous study)^33^ control sequences. There were also sequences with alanine scan (peptides **6**–**17**), C-terminus truncated sequences (**18**–**19**), N-terminus truncated sequences (**20**–**21**), and negatively (**22**–**23**) and positively (**24**–**27**) charged analogues. Finally the peptide library also had sequences with Y to F substitutions (**28**–**30**).

Peptide array on the cellulose membrane was used to evaluate the binding of each peptide to different cell lines. The amino functionalized cellulose membrane provided by Intavis is acid hardened providing high stability at pH range from 1–14, permitting its use in acidic or basic conditions while maintaining membrane integrity. The PEG linker on membrane surface functions as a hydrophilic spacer between the peptide and the solid surface (membrane) reducing background signal. Cell lines were carefully selected for screening peptides as our goal here is to screen peptides with high binding/uptake by the TNBC cells. Our lead peptide, peptide **1** (or GE11) targets and binds EGFR. EGFR is overexpressed by a number of TNBC cells such as MDA-MB-468 (obtained from primary tumor source) and MDA-MB-231 (from metastasis cancer tissue)^13,14^. While other TNBC cells like MDA-MB-436 (obtained from metastasis cancer tissue) are known to express low levels of EGFR. Non-tumorigenic epithelial MCF10A and MCF12A cells derived from the breast tissue also express low levels of EGFR^14,34^. We selected two TNBC cell lines with relatively high expression of EGFR expression (MDA-MB-468/231) and a TNBC cell line with low level of EGFR expression (MDA-MB-436) for the peptide array screening experiments. In addition, non- tumorigenic cell line, MCF-10A was used for comparison. These four cell lines were allowed to bind the membrane by seeding the cells on the membrane and the bound cells were labeled with a fluorescent dye CyQUANT.

### Peptide Library Screening

To screen the library and identify high binding peptides to TNBC cells, the relative cell adhesion was monitored by comparing the fluorescence of cells (cancer or non-cancerous) bound to wild type peptide **1** (Table 1). Peptides **22** and **23** displayed highest affinity (up to 2.8-fold compared to peptide **1**) to the three TNBC cell lines. These two peptides with Q9E and N10D substitutions, respectively, are negatively charged (net charge −1). Two other peptides that also showed high affinity were peptides **26** and **27**. Here again the two same amino acids (Q9 and N10) were substituted, however, with positively charged lysine. Analysis of the alanine scan showed that substitution of these two residues (Q9 and N10) with alanine (especially peptide **14** with Q9A substitution) also gives higher binding to cancer cells. These results suggest that substitution of Q9 with a negatively charged glutamic acid (E) leads to highest cancer cell binding. In addition, alanine scan suggested that W3 is important for cancer cell binding as substitution of this residue with alanine led to decrease in relative cell adhesion ratio (0.8–1.0). The positive control peptide **5** showed higher binding to TNBC cells compared to peptide **1** (1.3–1.8-fold), however, positive control peptide **4** did not show much improvement in binding based on the peptide array-whole cell binding assay. Based on these results, four peptides **22**, **23**, **26**, and **27** that displayed highest binding to the TNBC cells and did not show any substantial increase in affinity for the non-cancerous cells (MCF10A) were selected for cell uptake studies.

### *In Vitro* Cell Uptake of Soluble (Free) Peptides

Flow cytometry was employed to determine the cellular uptake of selected six peptides (**1, 4, 22**, **23**, **26**, and **27**). As opposed to the membrane bound (conjugated) peptide in library screening, the peptides used here were soluble (or free) FITC-labeled peptides. Peptides were labeled with FITC in the N-terminal and a β-alanine residue was used as a spacer between the FITC and the peptide sequence. The same three TNBC cells lines (MDA-MB-468, MDA-MB-231 and MDA-MB-436) used for peptide array screening were used here as well. Peptides **1** and **4** were used as controls. Peptide **1** is the lead EGFR binding peptide, whereas, peptide **4** binds keratin 1. Peptide **4** was included in this experiment to compare the uptake of this keratin 1 binding peptide with the EGFR binding peptides. Peptides **22**, **23**, **26**, and **27** were identified as the best EGFR binding peptides from the library screening.

**Figure 1:**
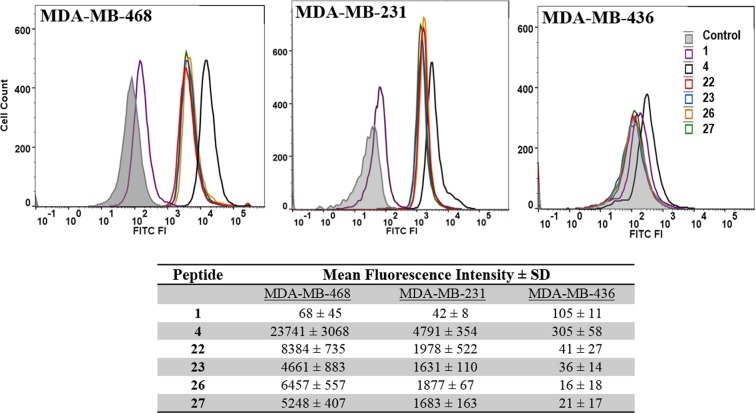
Uptake of the FITC-labeled peptides by triple negative breast cancer cells MDA-MB-468, MDA-MB-231, and MDA-MB-436. Cells (10^5^ or 10^6^/well) were treated with peptides (1 µM) and incubated for 30 min at 37 °C before analysis by FACS. The experiments were repeated twice and done in duplicates for each sample. Control group was treated with DMSO (5%). The table lists the mean fluorescence intensity (MFI) for the FITC positive cells for three independent experiments ± SD.

Figure 1 shows histograms and the mean fluorescence intensity (MFI) values for the three TNBC cell lines treated with six FITC-labeled peptides. Cell population treated with peptides **4**, **22**, **23**, **26**, and **27** showed significant FITC fluorescence in MDA-MB-468 and MDA-MB-231 when compared with cells treated with media only or treated with peptide **1**. Peptide **4** showed much higher uptake (MFI 23741) compared to the EGFR binding peptides (MFI 8384 or less). Among the new EGFR binding peptides, peptide **22** showed the highest uptake by the TNBC cells. In addition, the uptake of peptide **22** to MDA-MB-468 and MDA-MB-231 cells improved substantially with 123-fold and 47-fold higher affinity, respectively, compared to peptide **1**. The peptides showed highest uptake by the MDA-MB-468 cells followed by MDA-MB-231, and the lowest uptake was for MDA-MB-436 cells. Interestingly this is also the order of EGFR expression that is reported for these three cell lines with MDA-MB-468 showing highest level of EGFR expression^13,14^. The uptake of peptides **26** and **27** is only slightly less compared to peptide **22** even though these peptides have a substitution of a positively charged residue (K) for Q9 or N10 as opposed to negatively charged residue (E) for Q9 in peptide **22** (Fig. 1 table). It is anticipated that these peptide binds to the same site on EGFR as the native ligand EGF, and comparison of the amino acid sequence of these peptides and EGF shows that E and R may be preferred residues at positions 9 and 10 (vide infra).

In addition, it is notable that the uptake of the peptides by the cells is affected by the presence of free C-terminal carboxylic group, because the membrane bound peptides without free C-terminal carboxylic group showed less binding affinity to the cells compared to the soluble (or free) FITC labeled peptides which contain the free C-terminal carboxylic group. For instance peptide **22** showed only 2–3 fold improvement compared to peptide **1** (Table 1) using the peptide array-whole cell binding assay, whereas the uptake of peptide **22** by MDA-MB-468/231 cells is 50–123 fold compared to peptide **1** when evaluated using soluble (free) peptides in *in vitro* uptake using flow cytometry (Fig. 1).

### Specific Uptake of Peptides by TNBC Cells

**Figure 2:**
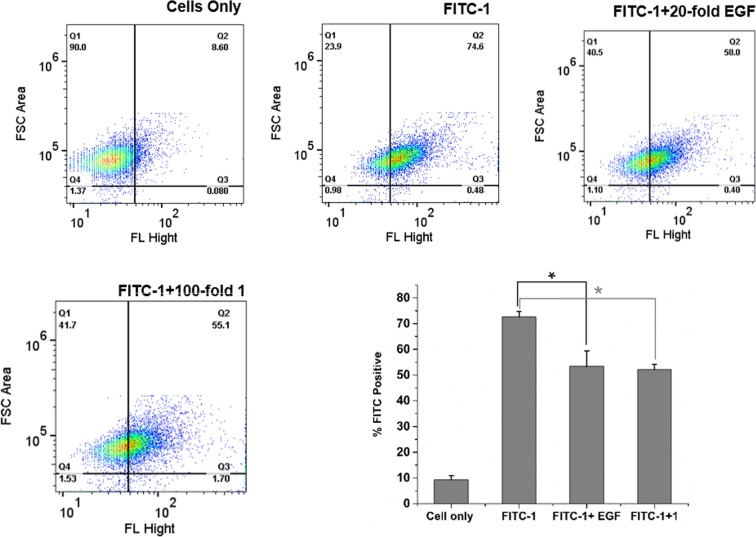
Competitive uptake of peptide **1** by FACS analysis. Fluorescence was measured for the MDA-MB-231 cells (10^5^ cells) alone or for the cells after incubation with peptide FITC-**1** (1 μM), peptide FITC-**1** in the presence of 20-fold excess EGF protein (20 μM) or peptide FITC-**1** in the presence of 100-fold excess unlabeled peptide **1** (100 μM). Also shown is the bar diagram for the percent of FITC positive cells in the absence and presence of excess free peptide **1** or EGF. Experiment was done in duplicates for each sample ± SD. The asteristik (*) denotes statistically significant deference (p < 0.05). Each well of cells was read twice in the FACS instrument. 10,000 cells were read.

**Figure 3:**
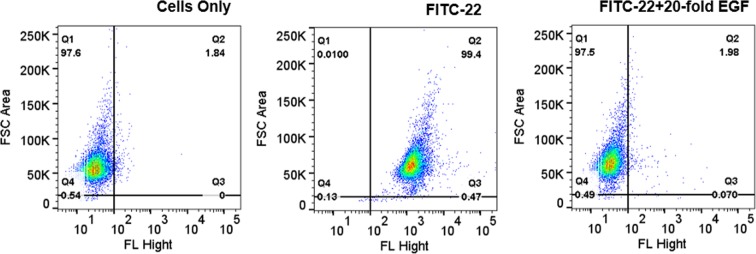
Competitive uptake of peptide **22** by FACS analysis. Fluorescence was measured for the MDA-MB-231 cells (10^5^ cells) alone or for the cells after incubation with peptide FITC-**22** (1 μM) or peptide FITC-**22** in the presence of 20-fold excess EGF protein (20 μM). Experiments were done in duplicates for each sample. 10,000 cells were read.

The specific uptake of peptides **1** and **22** was evaluated using a competitive assay. First, MDA-MB-231 cells were incubated with peptide FITC-**1** in the presence and absence of excess EGF protein (20-fold). The cells showed about 17% decrease in fluorescence when incubated in the presence of EFG protein (Fig. 2). Next, the cells were incubated with peptide FITC-**1** with or without excess unlabeled peptide **1** (100-fold). A similar decease in fluorescence (19.5% decrease) was observed when the cells were incubated with excess unlabeled peptide **1** suggesting multiple binding modes for peptide **1** on MDA-MB-231 cells. The competition experiment was also repeated with peptide **22** in the presence and absence of EGF protein. As shown in Fig. 3, the FITC positive cells reduced significantly from 99% to 2% (97% decrease) when the cells were incubated with peptide **22** in the presence of 20-fold excess EGF protein. The decrease in FITC positive cells was much higher for peptide **22** (97%) compared to peptide **1** (17%) suggesting that peptide **22** has higher specific binding to EGFR than peptide **1**. These results support our conjecture that the newly designed peptide **22** targets and binds EGFR, likely at the same site as EGF protein, and the binding or uptake can be reduced when the receptor is blocked with excess EGF.

Further we incubated a fixed number of MDA-MB-231 cells (10^5^) with different concentrations of peptide **22** (0–6 μM). The results showed that uptake was concentration independent in the range 1–6 μM (Fig. S1) suggesting that half saturation is below 1 μM. A direct interaction between the peptide and the isolated protein (EGFR) is required to confirm this specificity as well as the binding affinity. Previously Li *et al*. reported a *K*_d_ of 22 nM for peptide **1** binding to EGFR where the authors studied direct binding of the peptide to the pure EGFR protein^8^.

### Peptide 22 Structure

**Figure 4:**
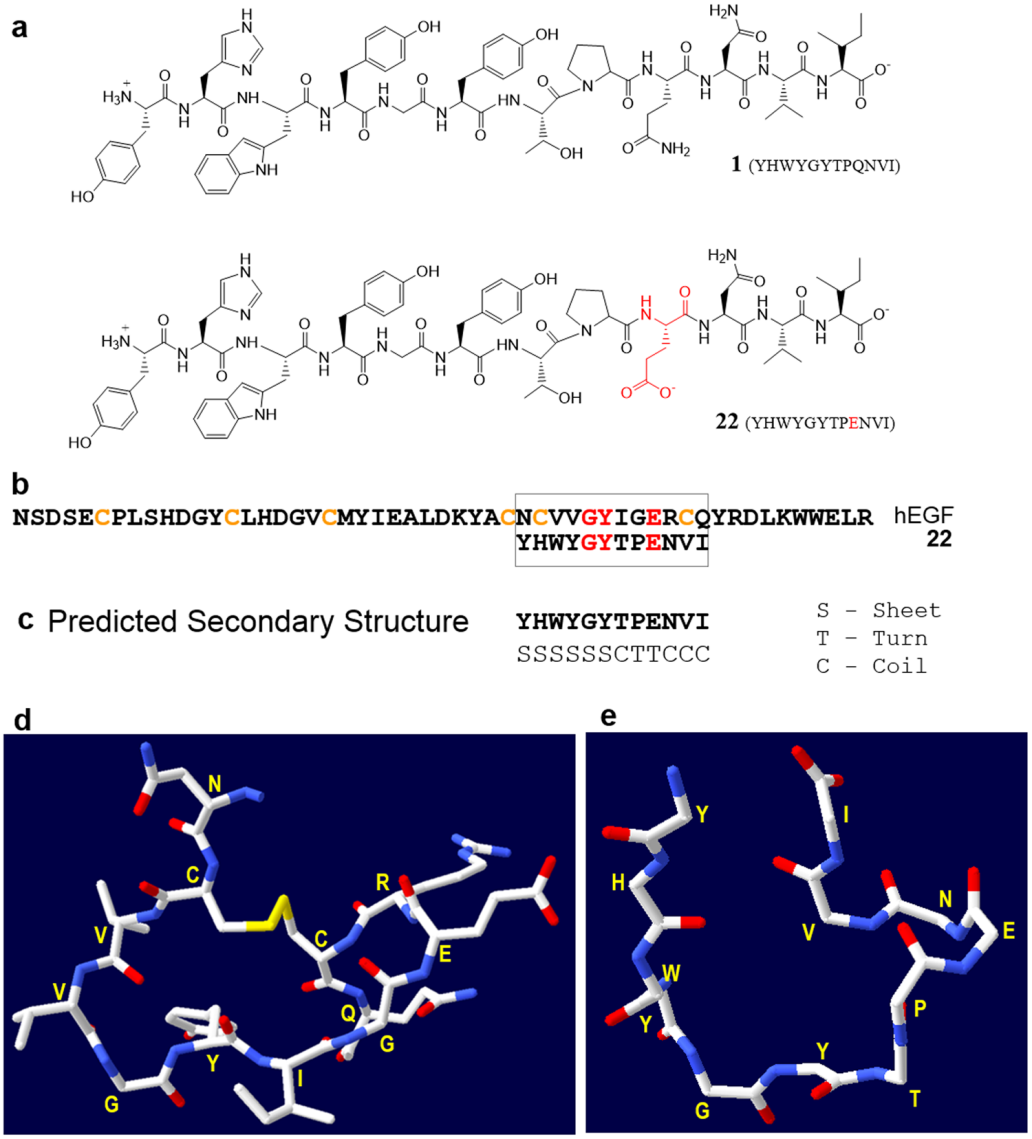
(**a**) Chemical structure of peptide **1** (GE11) and peptide analogue **22**. (**b**) Amino acid sequence of 53-mer human EGF (hEGF) and 12-mer **22** showing sequence homology for the G, Y and E residues (highlighted in red). Peptide **22** also shows structural similarity (based on secondary structure prediction of **22**) to this cyclic region of hEGF (residues 32–43). (**c**) Predicted secondary structure of peptide **22** using Chou and Fasman secondary structure prediction (CFSSP) server^37,38^. (**d**) 3D structure of a portion (residues 32–43) of hEGF derived from the crystal structure of full length hEGF (PDB code 1JL9)^35^. (**e**) Energy minimized structure of peptide **22** obtained using PEP-fold 2.0^43^ and Swiss-Pdb Viewer 4.1^42^. Side chains are not shown here for clarity.

As shown in Fig. 4a, the main difference in the chemical structures of peptides **1** and **22** is the presence of a negatively charged carboxylate in the side chain of glutamic acid of peptide **22**, whereas in peptide **1** this residue is uncharged (Gln). We next compared the sequence of peptide **22** with the EGF protein to understand the enhanced binding of peptide **22** to EGFR compared to peptide **1**. We found a region in EGF that may have structural similarity to peptide **22** (Fig. 4b). There is low sequence homology between the two sequences (peptide **22** and 12-residue region of EGF), however, based on the predicted secondary structure of peptide **22** and x-ray protein structure of EGF^35^ it seems that these two have high structural similarity. Notably, this region of EGF (32–43) contains several residues that are involved in direct interaction with the EGFR based on the site-directed mutagenesis studies as well as the crystal structure of human EGF in complex with EGFR extracellular region^36^. Residues 32–43 of EGF loop to form a cyclic structure (Fig. 4c) and secondary structure prediction^37,38^ of peptide **22** suggests that it consists of sheet and coil structures with a turn at P8 and E9 (Fig. 4d). Further, a homology based energy minimized structure for peptide **22** was obtained that shows structural similarly between residues 32–43 of EGF and peptide **22** (Fig. 4e). This structure displays that peptide **22** has a turn at E9 near the C-terminus and the peptide C-terminus coils back toward the N-terminus. These results suggest that a cyclic analogue of peptide **22** may give a high affinity EGFR binding peptide which should also have enhanced proteolytic stability. Such cyclic analogue of peptide **22** need to be tested. Peptide **22** shows short half-life (~1 hour) when incubated with human serum most likely due to proteolysis (data not shown). Previously we showed that a cyclic analogue (cyclic WXEAAYQkFL) of breast cancer targeting peptide **18–4** (WxEAAYQrFL, where lower case x is D-norleucine or D-Nle) was stable toward proteolytic degradation as well as displayed higher uptake by the breast cancer cells compared to the linear peptide^25^.

## Concluding Remarks

Four analogues (**22, 23, 26**, and **27**) of peptide **1** (YHWYGYTPQNVI) with high uptake by TNBC cells that express high levels of EGFR are reported in the current investigation. Among these four new analogues, peptide **22** showed the highest uptake by the TNBC cells. For both MDA-MB-468 and MDA-MB-231 cells, a 123-fold and 47-fold increase, respectively, in uptake of soluble FITC-labeled peptide **22** was observed compared to the lead peptide **1**. Both these cells lines (MDA-MB-468 and MDA-MB-231) are reported to express high levels of EGFR. Mueller *et al*. used lysates of breast cancer cell lines to obtain proteins which were separated by SDS-PAGE and immunoblotted with anti-EGFR to show overexpression of EGFR by these TNBC cell lines^14^. The specific binding and uptake of peptide **22** by EGFR in TNBC cells is further confirmed by competition assay where the uptake of **22** (99%) was reduced to 2% when the cells were incubated in the presence of excess EGF protein.

The comparison of peptide **22** sequence with a portion of EGF protein suggests that there may be structural similarity further supporting peptide’s specificity for EGFR. We are currently investigating an N- to C-terminal cyclic analogue of peptide **22**. Our preliminary results for the stability of peptides **1** and **22** in the presence of human serum show that these peptide have a short half-life (data not shown here). Our conjecture is that cyclization of peptide **22** will impart proteolytic stability while maintaining specificity for EGFR for its use in TNBC drug targeting. Targeted delivery of doxorubicin for the treatment of ovarian cancer was recently reported using peptide GE11 conjugated reversibly crosslinked polymersomes carrying doxorubicin^39^. These peptide GE11 conjugated polymersomes showed 2.5-fold higher accumulation in the tumor compared to liposomal doxorubicin, caused minimal side effects at high doses, and led to increased survival rate in SKOV3-tumor bearing mice^39^. The new analogues of peptide **1** (or GE11) such as peptides **22, 23, 26**, and **27** could serve as superior ligands for the targeted delivery of therapeutics for cancer treatment.

## Experimental Section

### Materials

All solvents and chemicals including triisopropylsilane (TIPS), dichloromethane (DCM), dimethylformamide (DMF), N-methylmorpholine (NMM), diethyl ether, ethanol, acetonitrile, trifluoroacetic acid (TFA) were obtained from Sigma-Aldrich (now Millipore Sigma, St. Louis, MO, USA). Piperidine (20%) was purchased from Protein Technology (AZ, Tucson, USA). CyQUANT and FITC dyes were obtained from Invitrogen (Eugene, Oregon, USA). Derivatized cellulose membranes were from Intavis (AG, Germany). Milli-Q system was used for ultrapure water. Media RPMI-1640 and Leibovitz’s L-15 were from American Type Culture Collection (ATCC, Manassas, VA, USA). DMEM, Step/Pen, Hank’s Balanced Salt Solution (HBSS), 0.05% trypsin/EDTA and horse serum were obtained from Gibco (USA) and Hyclone (Pittsburgh, PA, USA). Hydrocortisone was from Acros Organics (New Jersey, USA). Human recombinant EGF was obtained from Corning (New York, USA).

Peptide array syntheses were done on automated spot synthesizer ResPep SL apparatus (Intavis AG, Germany). Synthesis of soluble peptides was performed on automated peptide synthesizer Tribute from Protein Technologies (AZ, Tucson, USA). HPLC system used was Prominence-i Shimadzu (Kyoto, Japan). ChemiDoc™ XRS + system from Bio-Rad (California, USA) was used to record fluorescence intensity of spots on membranes. Cell uptake studies were carried out on BD FACSVerse Flow Cytometer (BD Biosciences, California, USA). Cells were counted using hemocytometer Neubauer Chamber. Peptide concentration was determined using Shimadzu BioSpec-nano Micro-volume UV-Vis Spectrophotometer (Shimadzu, Kyoto, Japan).

### Cell Lines

Cell lines were purchased from ATCC and were treated as recommended by ATCC for unpacking, handling, and culturing. MCF-10A and MCF-12A were cultured in DMEM/F12 media containing all components needed for growth media (Horse serum 5%, EGF 20 ng/mL, hydrocortisone 0.5/mL, insulin 10 µg/mL, Pen/Strep 1%). The media used for these two cell lines are modified version of ATCC. For culturing MDA-MB-436, L15 media supplemented with 10 µg/mL insulin, 16 µg/mL glutathione and 10% FBS was used. MDA-MB-231 and MDA-MB-468 were cultured in DMEM containing 10% FBS and 1% Pen/Strep. Except for MDA-MB-436, all cells were cultivated at 37 °C with 5% CO_2_. MDA-MB-436 cells were kept at 37 °C with 100% air. The conditions for cell culture for each of the cell lines are described in Supplementary Information Table S1.

### Peptide Array Synthesis

Thirty peptide sequences in duplicates were synthesized on functionalized cellulose membrane using fully automated ResPep SL peptide array synthesizer (Intavis, Germany) following previous procedure with some modifications (Fig. S2)^40,41^. Previously we used a semi-automatic AutoSpot (Intavis) synthesizer that required the fmoc deprotection with piperidine to be performed manually^32^. However, in the fully automated ResPep SL peptide array synthesizer all the steps for building the peptide chain are done by the robot. Briefly, the sequences of peptides were entered in robot software and 96 spot synthesis mode was selected for the library. The membrane was first immersed in DMF for an hour. It was then placed inside the holder of robot on a paper filter and was fixed with screws. The membrane was washed with ethanol few times and dried using vacuum to remove any bubbles below the membrane. Fmoc-peptide synthesis methodology with DIC and Oxyma pure as coupling reagent and racemization suppressor, respectively, was used. For each peptide spot, an 80 μL aliquot of activated amino acid (0.5 M) was delivered on the membrane surface using a robotic syringe. After each amino acid was double coupled, capping of unreacted amino acids was done using acetic anhydride (3%) and subsequently Fmoc-deprotection was achieved using 20% piperidine. After the synthesis was complete, the membrane was removed from the synthesizer platform and was manually dipped in deprotection solution containing approximately 50% TFA (15 mL), DCM (15 mL), TIPS (900 µL) and water (600 µL), and was kept for 3 h at room temperature^32^. The deprotection of the side-chain protecting groups was done using the same reaction conditions as originally developed. This procedure allowed complete removal of all side chain protections including the side chain Pbf of Arg^32^. Thereafter the membrane was washed extensively in several steps with DCM, DMF and ethanol, respectively. Finally it was air dried and kept in sealed bag at −20 °C until use.

### Peptide Array-Cell Binding Assay

For the binding assay, the peptide array was incubated with the cells in sequential steps as described previously^32^. Briefly peptide array membrane was dipped in ethanol for 30 s to remove any precipitation on it, followed by dipping in sterile PBS (pH 7.4) for another 30 min. The cells (75 × 10^3^ cells/mL) were seeded directly on the membrane in a sterile plate and incubated at 37 °C for 4 h. The unbound cells were washed out with sterile PBS (pH 7.4). Thereafter the membrane was placed in a sealed bag and transferred to −80 °C freezer for 2 h. Subsequently it was defrosted at room temperature and treated with CyQUANT solution. The CyQUANT concentration was optimized based on the manufacturer’s protocol and preliminary experiments. Membrane was incubated at 37 °C for 30 min with the dye. After washing with sterile PBS and air drying using blow dryer, the membrane was scanned using Bio-rad chemidoc imager with image lab software. The settings were adjusted based on the excitation (465 nm) and emission (535 nm) wavelengths of CyQUANT dye. Epi Blue was selected for the light source (455–485 nm) and filter number 4 (blue filter, 518–546 nm) was employed. The fluorescence intensity of cancer cells read using chemidoc imager was normalized using the non-cancer cell fluorescence intensity. Eventually relative fluorescence was assessed to select peptides with high affinity to cancer cells and low or minimal binding to normal cells.

### Synthesis of FITC Labeled Peptides

Six peptides, including peptide **1** (GE11), peptide **4** (p160) and analogues **22**, **23**, **26** and **27**, were selected for further studies. These peptides were synthesized following Fmoc solid phase peptide synthesis (Fmoc-SPPS) on preloaded Fmoc-isoleucine or leucine Wang resin (0.1 mmol scale) using automated peptide synthesizer (Tribute, Protein Technology) as reported previously. Preloaded Fmoc-isoleucine or leucine Wang resin (320 mg, 0.1 mmol) was added to the glass reaction vessel (RV) and the resin was allowed to swell in DMF under nitrogen with mechanical shaking for 30 min. All amino acids (3 equiv in 3 mL DMF) were coupled in sequence using HCTU (2.5 equiv) and NMM (1.2 equiv) for each coupling. Fmoc was removed by 20% piperidine in DMF. β-alanine was added at the N-terminal of each peptide sequence as a spacer before FITC coupling. FITC (0.3 mmol) in DMF (3 mL) with DIPEA (0.15 mmol) was added to the resin, and the mixture was incubated in the dark for 20 h. Small amount of the resin was used for a test cleavage using cleavage cocktail (1 mL) of trifluoroacetic acid (TFA)/triisoproylsilane/ ultra-pure water (95:2.5:2.5) for 2 hours. Cold diethyl ether (Et_2_O, 5 mL) was added to the filtered resin followed by centrifugation for 10 min in order to precipitate and collect the crude peptide. The successful conjugation was confirmed using MALDI-TOF and RP-HPLC of the test cleavage. Cleavage of FITC-peptides from the entire resin was done manually using same cleavage cocktail (10 mL) as described above. The peptides were precipitated using cold diethyl ether (20 mL). All peptides were characterized using MALDI-TOF mass spectrometry and RP-HPLC (Table S2 and Figs S3–S8). Purification was done using semi-preparative C18 Vydac column RP-HPLC.

### *In vitro* Cellular Uptake

Fluorescence-activated cell-sorting (FACS) analysis was used to check the uptake of the FITC-peptides. The stock solution for the FITC-peptides (100 μM) was prepared by dissolving the peptides in sterile water/10% DMSO. MDA-MB-231, MDA-MB-468 and MDA-MB-436 cells were seeded in 6-well plates with 10^6^/10^5^ cells/well containing 3 mL media and the plates were incubated at 37 °C overnight. The following day, the media was replaced with fresh FBS free media (1 mL) containing FITC-labeled peptide (1 µM), and further incubated at 37 °C for 30 min. Thereafter the media was removed and the cells were washed with cold PBS to remove any remaining peptide. Adequate trypsin was added to each well followed by 3–5 min incubation at 37 °C. Trypsin was immediately neutralized by adding media and the cell suspension was transferred into centrifuge tubes. The cells were separated and washed twice with FACS sheath fluid. The control cells were treated similarly but without peptide. Two wells were assigned for each peptide and each well sample was read twice in FACS machine. The experiment was repeated once. MFI represents the average of all wells for each peptide.

In addition, MDA-MB-231 cells (10^5^) were incubated with varying concentration of peptide FITC-**22** (0–6 μM). The resulting samples were treated as above, and the experiment was repeated. The fluorescence intensity of the FITC peptide-cell complex was plotted against peptide concentration. Finally, competition experiment was done in the presence of EGF (20 µM, 20x excess) or unlabeled peptide **1** (100 µM, 100 times more) as competitors. Protein EGF or unlabeled peptide was added to the wells first, followed by addition of FITC-labeled peptide **1** or peptide **22** and the plate was incubated for 30 min. Control group was treated with media alone containing ~5% DMSO. Each group was done in duplicate and the experiment was repeated once.

### Structure Prediction Methods

The Chou and Fasman secondary structure prediction (CFSSP) server was used to obtain predicted secondary structures for peptide **22**^37,38^. The three dimensional structure of a portion of EGF (12 residues, 32–43) was created using the PBD structure of EGF protein (PDB code 1JL9)^35^ and Swiss-Pdb Viewer 4.1^42^. A PDB structure for peptide **22** was generated using the de novo peptide structure prediction software PEP-fold 2.0^43^. The structure was further refined using the “magic fit” module and energy minimization of Swiss-Pdb Viewer 4.1^42^.

## Supplementary information

Supplementary Information
