# Supplementary material for: Small Peptide Ligands for Targeting EGFR in Triple Negative Breast Cancer Cells
Source: Sci Rep. 2019 Feb 25;9:2723. doi: 10.1038/s41598-019-38574-y (PMC6389950; doi:10.1038/s41598-019-38574-y)
Supplement: Supplementary file 1 — Supplementary Information [file 41598_2019_38574_MOESM1_ESM.pdf]

SUPPLEMENTARY INFORMATION

Small Peptide Ligands for targeting EGFR in Triple Negative Breast Cancer Cells

Hanieh Hossein-Nejad-Ariani, Emad Althagafi, and Kamaljit Kaur*

Chapman University School of Pharmacy (CUSP), Harry and Diane Rinker Health Science Campus, Chapman University, Irvine, California, 92618-1908, USA

Table of Contents

Figure S1. Uptake of peptide FITC-**22** to MDA-MB-231 cells by FACS................................S-2

Table S1. Cell culture conditions used for each cell line..S-3

Figure S2. An image of the peptide array on cellulose membrane S-4

Table S2. Amino acid sequences and characterization of FITC labeled cancer targeting peptides studied herein. S-5

Figure S3. RP-HPLC chromatogram and MALDI-TOF mass spectra of peptide FITC-1 ....S-6

Figure S4. RP-HPLC chromatogram and MALDI-TOF mass spectra of peptide FITC-4.........S-6

Figure S5. RP-HPLC chromatogram and MALDI-TOF mass spectra of peptide FITC-22 S-7

Figure S6. RP-HPLC chromatogram and MALDI-TOF mass spectra of peptide FITC-23 S-7

Figure S7. RP-HPLC chromatogram and MALDI-TOF mass spectra of peptide FITC-26 S-8

Figure S8. RP-HPLC chromatogram and MALDI-TOF mass spectra of peptide FITC-27 S-8

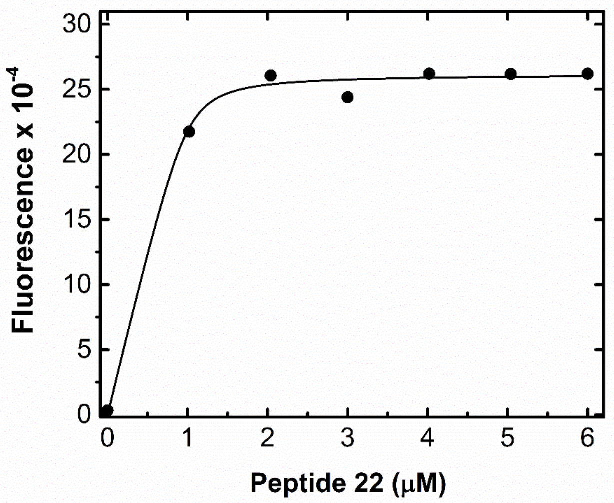

Figure S1. Uptake of peptide FITC-**22** to MDA-MB-231 cells by FACS. Cells (10^5) were treated with different concentrations of peptide (0, 1, 2, 3, 4, 5 and 6 μM) for 30 minutes at 37˚C. Experiment was done in duplicates for each concentration. The points are experimental and the line was calculated using a non-linear regression fit to the points.

Table S1. Cell culture conditions used for each cell line.

Cells	Growth media	Resuspension media
MDA-MB-231	DMEM/F12(+10% FBS+1% pen/step)	same as growth media
MDA-MB-468	Leibovitz's L-15 medium (+10% FBS+1% pen/step)	same as growth media
MDA-MB-436	Leibovitz's L-15 medium (+10 µg/ml insulin, 16 µg/ml glutathione+10% FBS+1% pen/step)	same as growth media
MCF-10A	DMEM/F12 (+5% Horse serum, EGF, hydrocortisone, insulin, 1% pen/step)	DMEM/F12 (+20% Horse serum, 1% pen/step)

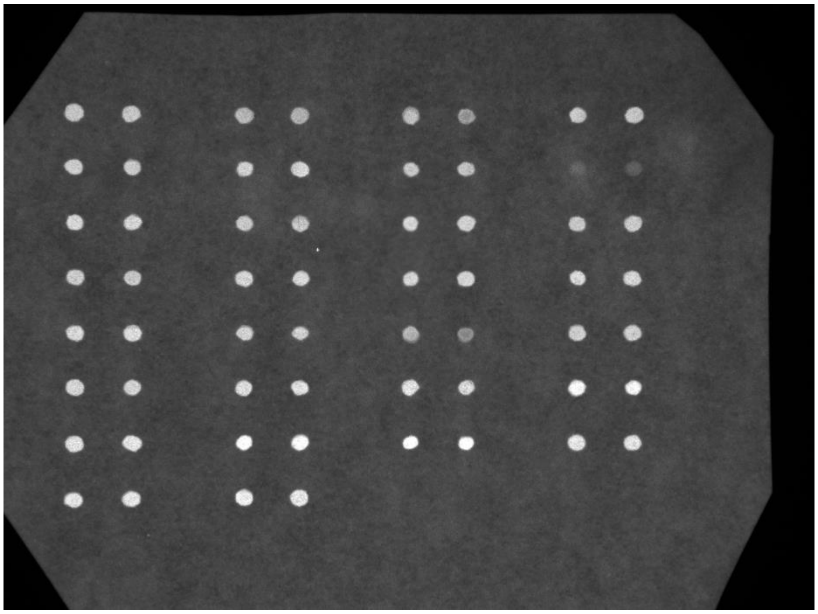

Figure S2. An image of the peptide array on cellulose membrane obtained using BioRad Chemidoc (light source Epi blue and blue emission filter) showing 30 peptides in duplicates. The first rows shows spots for peptides 1-4 in duplicates, second rows shows peptides 5-8, and so on.

Table S2. Amino acid sequences and characterization (MALDI-TOF mass spectrometry and RP-HPLC retention time) of FITC labeled cancer targeting peptides studied herein.

Peptide	Label	MW $[M+H]^{+1}$ Calculated	Found	Retention Time (min)
FITC-A_{β}YHWYGYTPQNVI	**1**	2001.1	2023.4 $[M+Na]^{+1}$	50
FITC-A_{β}VPWXEPAYQRFL	**4**	1980.1	1979.1 $[M+H]^{+1}$	58
FITC-A_{β}YHWYGYTPENVI	**22**	2002.1	2002.3 $[M+H]^{+1}$	50
FITC-A_{β}YHWYGYTPQDVI	**23**	2002.1	2023.7 $[M+Na]^{+1}$	50
FITC-A_{β}YHWYGYTPKNVI	**26**	2001.2	2023.0 $[M+Na]^{+1}$	49
FITC-A_{β}YHWYGYTPQKVI	**27**	2015.2	2015.7 $[M+H]^{+1}$	49

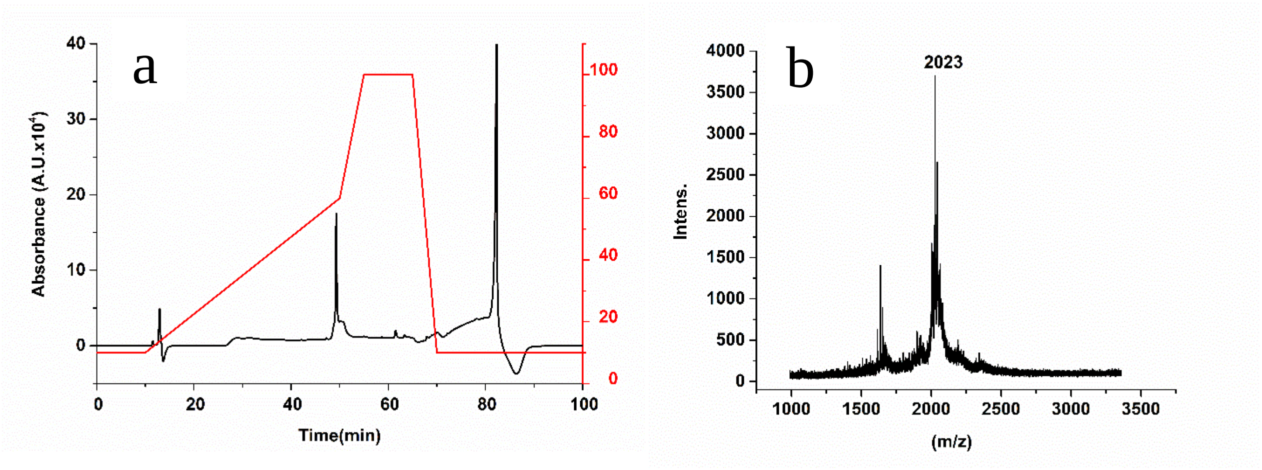

Figure S3. (a) RP-HPLC chromatogram of peptide FITC-**1**. The HPLC method used was 10-60% acetonitrile/water in 40 min with a flow rate of 1 mL/min on Vydac C18 semi-preparative column. (b) MALDI-TOF mass spectra of peptide FITC-**1** showing the $[M+H]^{+1}$ as the major peak.

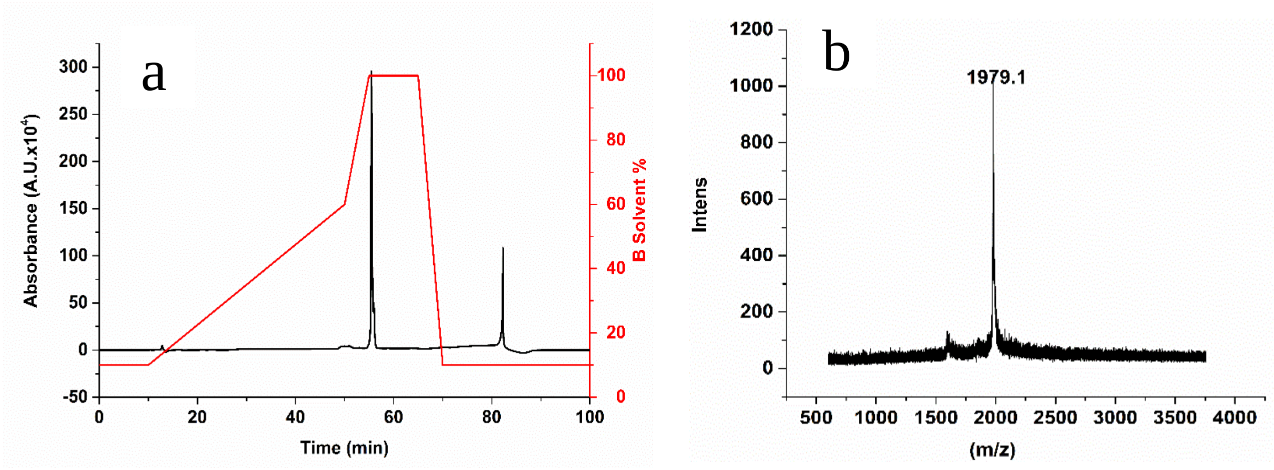

Figure S4. (a) RP-HPLC chromatogram of peptide FITC-**4**. The HPLC method used was 10-60% acetonitrile/water in 40 min with a flow rate of 1 mL/min on Vydac C18 semi-preparative column. (b) MALDI-TOF mass spectra of peptide FITC-**4** showing the $[M+H]^{+1}$ as the major peak.

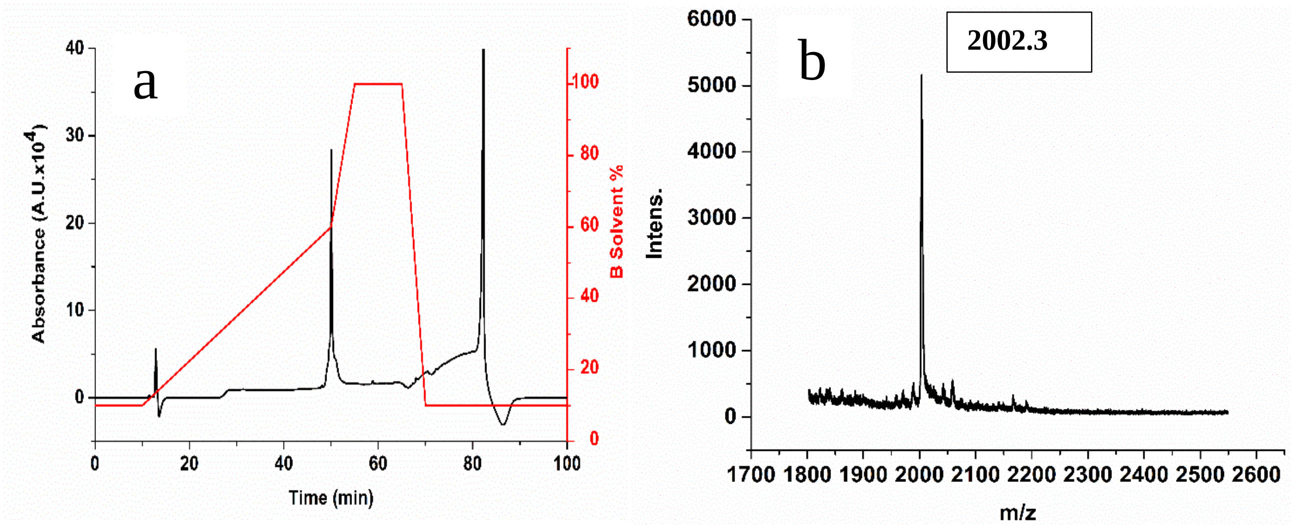

Figure S5. (a) RP-HPLC chromatogram of peptide FITC-**22**. The HPLC method used was 10-60% acetonitrile/water in 40 min with a flow rate of 1 mL/min on Vydac C18 semi-preparative column. (b) MALDI-TOF mass spectra of peptide FITC-**22** showing the $[M+H]^{+1}$ as the major peak.

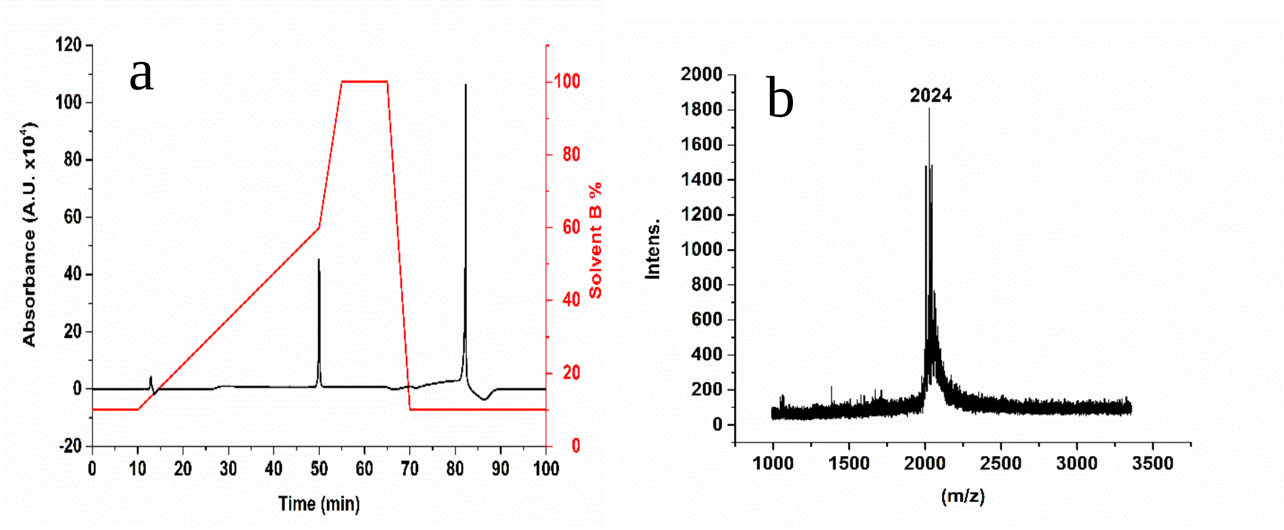

Figure S6. (a) RP-HPLC chromatogram of peptide FITC-**23**. The HPLC method used was 10-60% acetonitrile/water in 40 min with a flow rate of 1 mL/min on Vydac C18 semi-preparative column. (b) MALDI-TOF mass spectra of peptide FITC-**23** showing the $[M+H]^{+1}$ as the major peak.

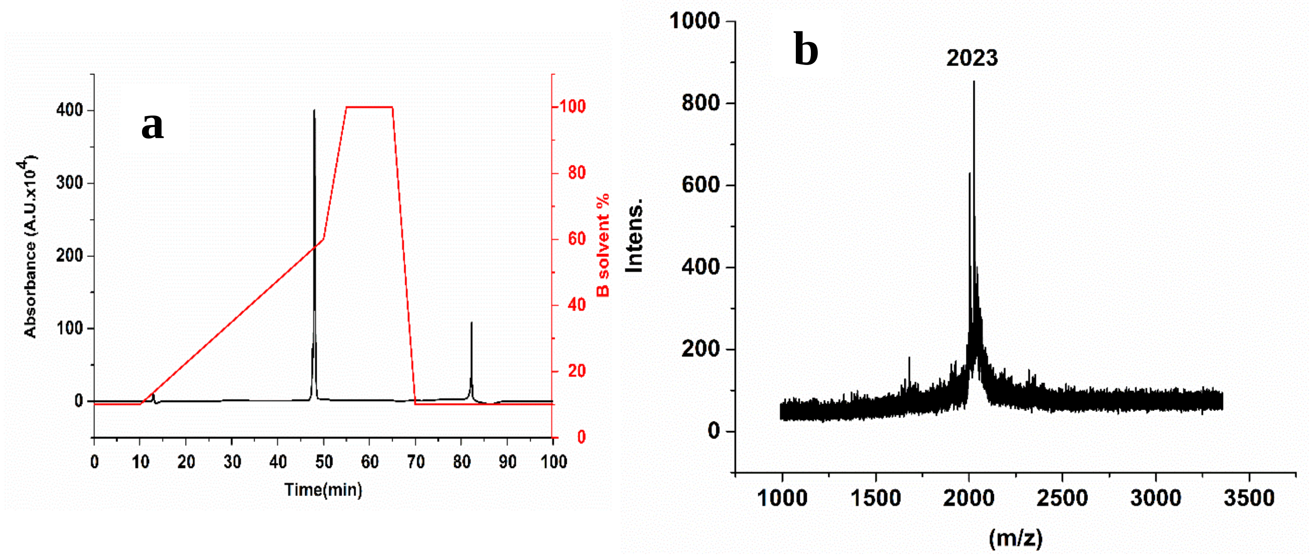

Figure S7. (a) RP-HPLC chromatogram of peptide FITC-**26**. The HPLC method used was 10-60% acetonitrile/water in 40 min with a flow rate of 1 mL/min on Vydac C18 semi-preparative column. (b) MALDI-TOF mass spectra of peptide FITC-**26** showing the $[M+H]^{+1}$ as the major peak.

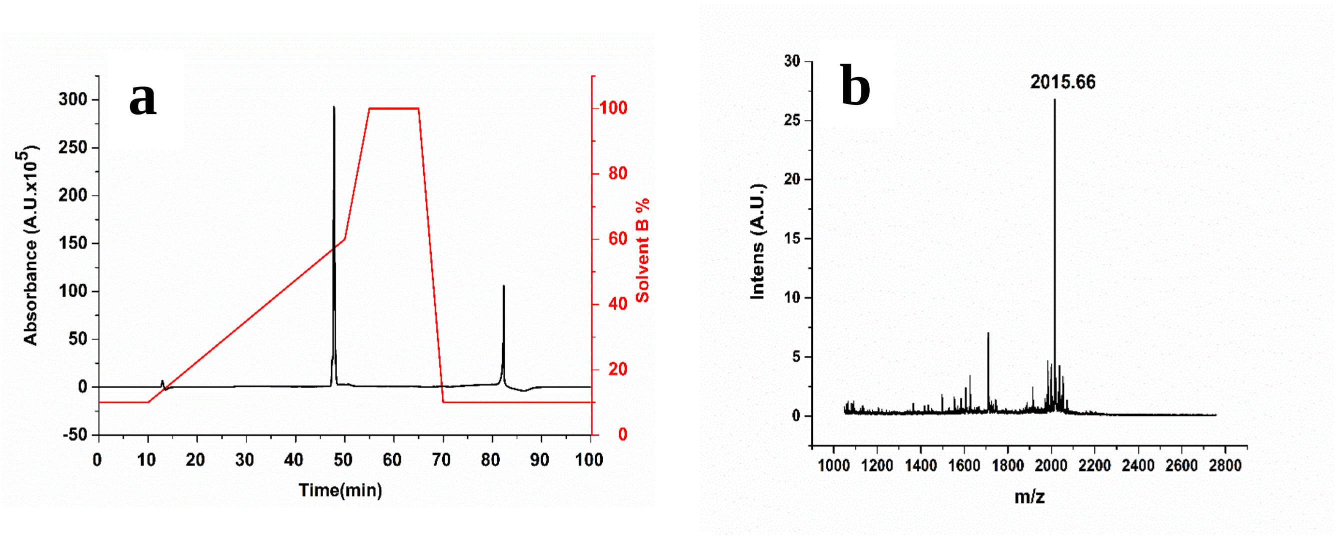

Figure S8. (a) RP-HPLC chromatogram of peptide FITC-**27**. The HPLC method used was 10-60% acetonitrile/water in 40 min with a flow rate of 1 mL/min on Vydac C18 semi-preparative column. (b) MALDI-TOF mass spectra of peptide FITC-**27** showing the $[M+H]^{+1}$ as the major peak.
